# Molecular Mechanisms of *Nigella sativa*- and *Nigella sativa* Exercise-Induced Cardiac Hypertrophy in Rats

**DOI:** 10.1155/2021/5553022

**Published:** 2021-04-09

**Authors:** Lubna Ibrahim Al Asoom

**Affiliations:** Physiology Department, College of Medicine, Imam Abdulrahman Bin Faisal University, Dammam, Saudi Arabia

## Abstract

**Background:**

In our lab, we demonstrated cardiac hypertrophy induced by long-term administration of *Nigella sativa* (Ns) with enhanced function. Therefore, we aim to investigate the molecular mechanisms of Ns-induced cardiac hypertrophy, compare it with that induced by exercise training, and explore any possible synergistic effect of these two interventions.

**Method:**

Twenty adult Wistar male rats were divided into control (C), Ns-fed (N.s.), exercise-trained (Ex.), Ns-fed exercise-trained (N.s.Ex.) groups. 800 mg/kg of Ns was administered orally to N.s. rats. Ex. rats were trained on a treadmill with speed 18 m/min and grade 32° for two hours daily, and the N.s.Ex. group underwent both interventions. After 8 weeks, Immunohistochemical slides of the left ventricles were prepared using rat growth hormone (GH), insulin-like growth factor I (IGF-I), angiotensin-II receptors 1 (AT-I), endothelin-I (ET-1), Akt-1, and Erk-1. Cell diameter and number of nuclei were measured.

**Results:**

Cardiomyocyte diameter, number of nuclei, GH, and Akt were significantly higher in N.s, Ex., and N.s.Ex groups compared with the controls. IGF-I, AT-1, and ET-1 were significantly higher in Ex. rats only compared with the controls. Erk-1 was lower in N.s., Ex., and N.s.Ex. compared with the controls.

**Conclusion:**

We can conclude that Ns-induced cardiac hypertrophy is mediated by the GH-IGF I-PI3P-Akt pathway. Supplementation of Ns to exercise training protocol can block the upregulation of AT-I and ET-1. The combined N.s. exercise-induced cardiac hypertrophy might be a superior model of physiological cardiac hypertrophy and be used as a prophylactic therapy for athletes who are engaged in vigorous exercise activity.

## 1. Introduction

Cardiac hypertrophy is an adaptive response of the heart to an increase in the volume or stress load. It is characterized by an increase in the thickness of the left ventricle that is attributed to the enlargement of the cardiomyocytes [1]. Clinical and experimental observations have identified two forms of cardiac hypertrophy: the first one is precipitated by pathological insults such as stress overload due to hypertension and valvular heart disease or injury of the cardiac muscle due to ischemia and infarction, whereas the other one is developed as a consequence of volume and stress overload induced by chronic exercise training [2]. The former cardiac hypertrophy is recognized as pathological cardiac hypertrophy. Mostly, the initial adaptive responses to a pathological insult are brought about to enhance the cardiac contractility and maintain the functional requirement of the heart. However, as the insult persists, it overwhelms some of the compensatory pathways and allows the deviation into undesirable changes. Consequently, the ultimate encountered cardiac remodeling precipitates multiple faulty structural, electrical, and biochemical modifications that downgrade the function of the heart [3].

On the other hand, cardiac hypertrophy initiated by volume overload of physiological origin such as exercise training maintains optimum cardiac function and provides favorable multiple structural, electrical, and biochemical remodeling that enhanced the cardiac function [4].

Two distinguished molecular pathways were recognized for each of the previously described cardiac hypertrophy. The pathological hypertrophy is mediated through the G-protein-coupled receptor (GPCR) pathway. The GPCR main precursors are angiotensin-II, endothelin-1, and catecholamines. The intracellular cascades involve multiple mitogen-activated protein kinase MAPK enzymes such as Erk1/2 [5]. On the other hand, the physiological cardiac hypertrophy is mediated via the activation of tyrosine kinase receptors by growth hormone (GH) and insulin-like growth factor I (IGF-I) that culminates in triggering the phosphoinositide 3-kinase–RAC-*α* serine/threonine-protein kinase enzyme and Akt (PI3K–AKT) [6].

The physiological cardiac hypertrophy induction was implemented by some studies as therapeutic trials for the decompensated pathological hypertrophy. Favorable adaptation and switch to compensatory pathways were observed in some experimental animals. However, clinical evidence is not sufficiently reported. Weak compliance toward following different exercise regimens particularly by the cardiac patients hinders many clinicians and researchers from reporting promising outcomes [7].

*Nigella sativa* (Ns), which is a traditional remedy used by multiple countries in the Middle East, was found to induce cardiac hypertrophy in Wistar rats after 8 weeks of oral administration [8]. The structural, functional, and electrophysiological features of the Ns-induced cardiac hypertrophy were found normal and comparable to that induced by exercise training [9, 10]. Therefore, it was postulated that long-term administration of Ns can generate physiological cardiac hypertrophy. To confirm this hypothesis, it would be necessary to identify the mediating molecular pathway. Therefore, in the current study, we aim to estimate the major molecular elements involved in both pathological and physiological pathways and compare it between exercise-training-induced cardiac hypertrophy as a known model of physiological cardiac hypertrophy and Ns-induced cardiac hypertrophy, that is, our model of interest.

## 2. Materials and Methods

This experimental work was approved by the Institutional Ethical Committee of Imam Abdulrahman Bin Faisal University, Dammam, Saudi Arabia with IRB number 2020-01-263. The minimal required number of adult healthy rats (200–300 gram in weight) was obtained from the animal house of Imam Abdulrahman Bin Faisal University. Each 5 rats were assigned for one of the following groups: control, *Nigella sativa*-fed (N.s.), exercise-trained (Ex.), and Ns-fed exercise-trained (N.s.Ex.). The rats were placed in individual labeled cages. The environment was controlled with adequate ventilation and illumination and normal light cycle (12 light/12 dark); *ad libitum* access to normal laboratory chow and tap water was ensured [11].

The N.s. rats received daily dose of 800 mg/kg of body weight in the form of oral solution of freshly ground N.s. for eight weeks. Details related to the preparation of the dose and the feeding technique are described in our previous studies [12]. The black seeds (N.s.) used in this study are a harvest of the Qassim area in the central province of Saudi Arabia. The constituents of this type of black seeds were reported by Al-Jassir [13]. The control rats were fed with an equivalent volume of water.

The rats in the Ex. group underwent a training on a treadmill (IITC Life Science; five-lane rat treadmill), five days/week for eight weeks. A progressive increment in the speed, grade, and duration was adjusted during the first week until the target protocol was achieved, with a speed of 18 m/min and an inclination of 32°, for a two-hour session/day [14]. An equivalent volume of water was also fed to this group. The fourth group (N.s.Ex.) exposed to both interventions, i.e., Ns feeding and exercise training.

### 2.1. Extraction of the Hearts

After anesthetizing the rats with intraperitoneal injections of 0.2 mL/250 g body weight of ketamine cocktail (60% ketamine, 40% xylazine), the hearts were dissected, cleaned from connective tissue, rinsed, and impeded in cold Ringer's solution. Then, the hearts blotted dry and weighed. Similarly, the left ventricles were dissected and weighed. The free wall of the left ventricles was extracted and stored in 4% formal saline for histological preparation.

### 2.2. Preparation of the Light Microscopic Slides

After fixation of the left ventricular wall in 4% formal saline, the specimens were washed shortly in water, labeled, and incubated in a tissue processor (Tissue-Tek VIP) overnight. The specimens were dehydrated in the following concentrations of alcohol—70%, 90%, and 100% two changes—and two changes of xylene for a period of two hours, respectively.

Then, the process of embedding was started by impregnating the specimens in two changes of molten paraffin wax for a period of two hours for each change and one at a temperature of 60°C. The position of the specimens was controlled by cassettes. The prepared blocked tissues were labeled and allowed to solidify. They were then sectioned using microtomy (LEICA RM 2235; Leica BioSystems, Buffalo Grove, IL, USA) at a thickness of 3 *μ*m. The sectioned tissues were floated in warm water and then placed on microscope slides, labeled, and allowed to dry. The sections were dewaxed, washed in water, and stained with hematoxylin & eosin (H&E) or using the following antibodies:

1. Anti-growth hormone (mouse/rat growth hormone biotinylated affinity purified, goat IgG, R&D system, USA)
2. Anti-IGF-I antibodies (from Sigma-Aldrich for immunohistochemistry, USA)
3. Anti-angiotensin-II receptor type I (AT1) antibodies (from Sigma-Aldrich, USA)
4. Anti-endothelin-I antibodies (ET-1) (from Sigma Aldrich, USA)
5. Phospho-Akt (S473) pan-specific affinity purified PAb, rabbit IgG (Biocompare)
6. Phospho-Erk1 (T 202/Y204) Erk2 (T185/Y187) affinity purified PAb, Rabbit IgG (Biocompare)

### 2.3. Estimation of the Antibody Labeling in the Light Microscopic Slides

The immunohistochemical slides of the left ventricular wall were captured using a digital microscope (Coolscope; Nikon Instruments Europe BV, Amsterdam Netherlands). The fields were selected carefully to avoid overlap, freezing defect, gaps, or folds. Ten fields were obtained per specimen per stain or antibody under a power of X400. The photomicrographs were studied using an Image J analyzer. The cell diameter was considered the length of the vertical line connecting the cell membranes of the cardiomyocyte and crossing the nucleus. The diameter of 100 cells per specimen was measured, and then the average was calculated. The number of nuclei was counted manually in 10 fields per specimen. For assessing the immune-histochemical slides, the stained area was estimated and then expressed as a ratio of the stained area to the total field area.

**Figure 1:**
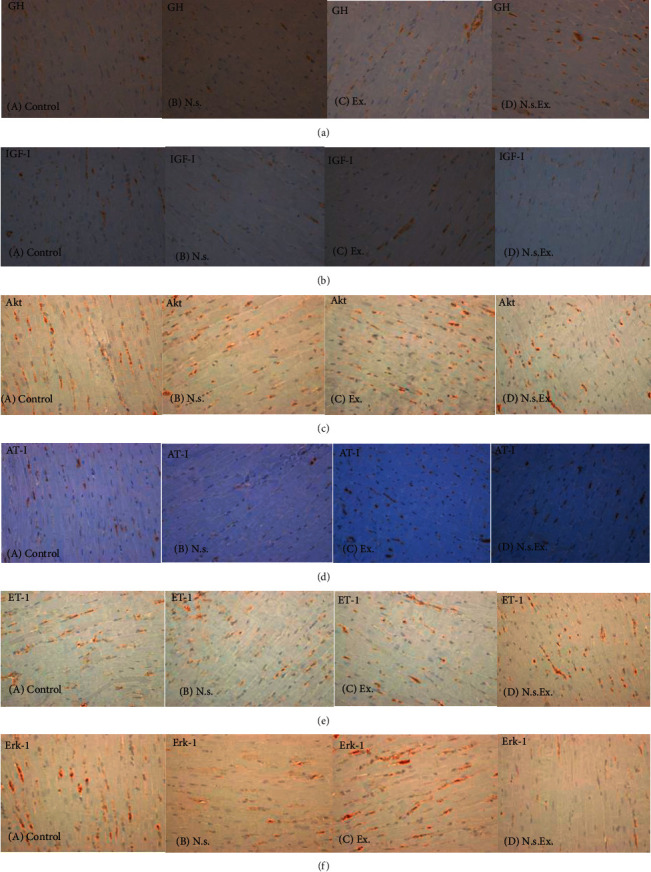
Photomicrographs of the immunohistochemical slides of the left ventricular wall stained with (a) GH, (b) IGF-I, (c) Akt, (d) AT-1, (e) endothelin-1, and (f) Erk-1 obtained from a. Control, b. N.s., c. Ex., and d. N.s.Ex. rats. N.s.: *Nigella sativa*-fed group; Ex.: exercise-trained group; N.s.Ex.: *Nigella sativa*-fed exercise-trained group; GH: growth hormone; IGF-I: insulin-like growth factor-I; Akt: also known as protein kinase B (PKB); AT-1: angiotensin-II receptor type 1; ET-1: endothelin-1; Erk1/2: extracellular signal-regulated kinases ½.

The mean value of the immunostained area for all fields of a specific antibody in each group was obtained (see Figure 1 for the photomicrographs of all antibodies).

### 2.4. Statistical Analysis

The data were analyzed using IBM—Statistical analysis software package—SPSS, version 20. All data were expressed as mean ± SD and tested by analysis of variance (ANOVA) and LSD post hoc tests to detect any difference between the groups. The level of significance was set at *p* < 0.05.

## 3. Results

Photomicrograph samples representing the immunohistochemical slides of each antibody obtained from each group are demonstrated in Figure 1. The mean value of the cardiomyocyte diameter, the number of nuclei, and the percentage of the stained areas were compared between all the groups, i.e., the control, N.s., Ex., and N.s.Ex. groups using ANOVA and LSD post hoc tests, and the data are presented in Table 1. Cardiomyocyte diameter, number of nuclei, GH, and Akt were significantly higher in all the experimental groups—N.s., Ex., and N.s.Ex. groups—than the control group. While IGF-I, angiotensin-II receptor type 1 (AT-1), and endothelin-1 (ET-1) were significantly higher in Ex. rats only compared with the control group. Erk1/2 was significantly lower in all experimental groups compared with the control group.

### 3.1. Histopathological Examination

Histopathological examination of H&E-stained sections of the left ventricular tissue of all the groups revealed a comparable normal structure and integrity of the cardiomyocytes. The cardiomyocytes appeared in these sections faintly striated and multinucleated. There were no signs of inflammation or collagen fiber deposition in any of the experimental sections compared with the control apart from the increase in cardiomyocyte diameter and number of nuclei stated earlier in the previous paragraph.

## 4. Discussion

The present study aims to investigate the molecular mechanisms underlying the Ns-induced cardiac hypertrophy and compares it to the mechanisms of exercise-induced cardiac hypertrophy. Concurrently, the synergistic effect and the interaction of the underlying molecular mechanisms of both Ns administration and exercise training are also sought. The cardiac hypertrophy induced by Ns was demonstrated earlier in our lab [9] by an increase in the ratio of the heart weight/bodyweight and left ventricular weight/bodyweight and the higher cardiomyocyte diameter of the N.s. rats and Ex. rats compared with controls. Similarly, we were able to demonstrate in this current work a comparable significant increase in the cardiomyocyte diameter in N.s. rats, Ex. rats, and N.s. Ex rats compared with the controls, as well as we found for the first time a significant increase in the number of the cardiomyocyte nuclei in the microscopic sections of the left ventricular wall of all the experimental groups compared with the controls. This latter finding could be an additional evidence of the growth of the cardiac muscle by our current experimental interventions. Bruel et al., who demonstrated about 31% increase in the number of cardiomyocyte nuclei in the GH-treated rats compared with the controls, proposed that the increase in the number of the cardiomyocyte nuclei upon GH administration to young rats is one of the signs of cardiac growth and might be a product of cardiomyocyte hyperplasia [15, 16]. However, other authors requested additional advanced imaging and labeling techniques for the nuclei to confirm its status and to demonstrate signs of mitosis that affirm the cell division [17].

On the other hand, exploring the underlying molecular mechanisms yielded an evidence of an increment in the GH-IGF I-PI3K-AKT1 pathway, which was manifested specifically by the significant increase in GH and the subcellular enzyme Akt but without evidence of a significant rise in IGF-1. This pathway was frequently affirmed as the main pathway responsible for mediating the physiological cardiac hypertrophy induced by exercise training [18, 19]. The effect of GH, per se, in the induction of physiological cardiac hypertrophy was demonstrated by Fu et al. [20] in transgenic mice with overproduction of GH, which exhibited growth and hypertrophy of the cardiomyocytes with normal alignment of the myofibrils and normal structure of the organelles. In addition, administration of GH to rats enhances the performance of the heart, the strength of contractility, and the growth of the myocardium without evidence of fibrosis [21, 22].

GH action is mediated via the release of IGF-I from various tissues such as the liver, the myocardium, and the skeletal muscle. IGF-I, which is a peptide similar to insulin in structure, is released in the myocardium in a paracrine mode, binds to transmembrane tyrosine receptors in the cardiomyocytes, and provokes the activation of PI3K-Akt pathway to mediate the hypertrophy of the heart [23]. Experimentally, the application of IGF-I on a culture of adult cardiomyocytes induces hypertrophy of the cells, increases myofibril proteins, promotes the synthesis of new sarcomeres, and upregulates its receptors [24–26]. Administration of GH, IGF-I, or a combination of both factors to rats or mice elicited concentric cardiac hypertrophy manifested by an increase of the cardiac index and the myocyte diameter without significant increase in fibrosis, in addition to an increase in the myocardial contractility [22, 27]. In our study, IGF-I was significantly elevated in the Ex. group, whereas it was equivalent to the controls in N.s. and N.s.Ex groups despite the documented cardiac hypertrophy in these groups too. As a possible explanation, we might propose that Ns administration might culminate in the upregulation of another analogue of IGF-I to mediate the action of the pathway GH-IGF I-PI3K-Akt. We might rely in our proposal on the notion that the IGF-I superfamily (IGF-I, IGF-II, insulin) possesses a great overlap and crosstalk in both the function and the structure [28]. On the other hand, some evidence in the literature showed that a high IGF-I level might not be a necessity to induce cardiac hypertrophy by the GH-IGF I-PI3K-Akt pathway, as multiple experimental animal studies showed conflicting findings in regard to the level of IGF-I after a period of training. Some studies showed high cardiac IGF-I, but others failed to demonstrate any increment in this growth factor despite the documented increase in cardiac size. Kim et al. pointed toward the significance of the IGF-I receptors and its upregulation upon training of normal rats and IGF-I receptor knockout rats in mediating cardiac hypertrophy [29, 30].

Akt is the subcellular enzyme involved in the GH-IGF I-PI3K-Akt pathway. Akt is found higher in all the experimental groups than the controls. Profound evidence indicates the significance of Akt for the development of the physiological cardiac hypertrophy and not the pathological. This was demonstrated when transgenic mice that lack Akt were subjected to swimming and aortic ligation; the first group failed to develop cardiac hypertrophy, whereas the second one with aortic ligation manifested an increase in the heart weight compared with normal controls, which underwent the same interventions [23].

Furthermore, while exploring the response of the GPCR pathway through the expression of AT-1, ET-1, and the subcellular enzyme Erk1/2, we found that AT-1 and ET-I were significantly elevated in the Ex. group only compared with the control group, whereas Erk1/2 was significantly reduced in all the experimental groups (N.s., Ex., and N.s.Ex.) compared with the control group. Referring to our earlier discussion about the role of GPCR pathway in mediating the pathological cardiac hypertrophy, we found that the significant high levels of AT-1 and endothelin-1 appear, for the first glance, conflicting with the literature data. However, despite the knowledge that exercise training induces physiological cardiac hypertrophy via the GH-IGF-I-PI3K-Akt pathway, some reports revealed possible decompensation of the exercise-induced cardiac hypertrophy and deterioration of the cardiac function in competitive sport athletes [31]. Nevertheless, sudden cardiac arrest and fetal arrhythmias are often encountered in long-distance runners [32]. Vigorous or prolonged exercise activity frequently incurs the activation of pathological cardiac pathways and yields elevated biomarkers of cardiac damage [33]. Furthermore, some competitive long-distance runners developed exercise-induced hypertension, which was associated with an abnormal level of angiotensin-II [34]. ET-1 was also found elevated significantly in the Ex. group compared with controls. ET-1 might also contribute to the conversion of the exercise-induced cardiac hypertrophy into pathological remodeling at extensive strenuous exercise. Iemitsu et al. reported high ET-1 levels in rats after 8 weeks of treadmill training and not after 4 weeks of training [35]. However, there is no clear determination of the period or the intensity of exercise training that is associated with the eliciting of the pathological pathways of angiotensin-II or ET-1. Interestingly, angiotensin-II and ET-1 were not elevated in the N.s.Ex. group, which underwent the same exercise protocol as that of the Ex. group. This finding could pinpoint a beneficial adaptive response of the long-term administration of Ns through its ability to block the exercise-induced upregulation of AT-1 and ET-1. It may also reflect that the Ns-induced cardiac hypertrophy is a safer and healthier model than that induced by exercise training. Therefore, Ns supplements might be recommended for cardiovascular protection particularly for vulnerable candidates such as those involved in highly competitive sports.

Erk1/2 was found reduced in all the experimental groups compared with controls. The reduction of Erk1/2 was more pronounced in the N.s.Ex. group. The current finding is in accordance with the proposal that both Ns- and exercise-induced cardiac hypertrophies are physiological forms of cardiac hypertrophy. The reduction of Erk1/2 was also reported in the hippocampal tissue of trained rats [36]. However, since our trained rats demonstrated an increase in AT-1 and endothelin-1, then the reduction in Erk1/2 might be visualized as an unexpected finding. Nevertheless, the subcellular molecular cascade of GPCR is complex and includes multiple parallel intermediate kinases besides Erk1/2. Therefore, the downregulation of Erk1/2 might be associated with the upregulation of another kinases. Undoubtedly, these postulations might not be confirmed without a comprehensive analysis of all possible alternative pathways.

## 5. Conclusion

We conclude from the findings of the present study that Ns administration for 8 weeks can induce physiological cardiac hypertrophy manifested by an increase in the cardiomyocyte diameter and the number of cardiomyocyte nuclei. This type of cardiac hypertrophy is mediated via the GH-IGF I-PI3K-Akt pathway as reflected by the significant elevation of GH and Akt. Furthermore, the adjuvant supplementation of Ns to exercise training protocol hinders the adverse responses generated by vigorous exercise and blocks the undesirable provocation of the pathological pathways of GPCR. Therefore, besides the Ns-induced cardiac hypertrophy, we can present in this study a new superior model of physiological cardiac hypertrophy by the synergistic favorable effects of both Ns feeding and exercise training. The N.s. exercise-trained cardiac hypertrophy model can be recommended as a prophylactic strategy for vulnerable individuals such as elite athletes.

## Figures and Tables

**Table 1:** Comparison of the histological and the immunohistochemical findings of the left ventricular walls among the experimental groups using ANOVA and LSD post hoc test.

	Control	N.s.	Ex.	N.s.Ex.
Cell diameter	88.8 ± 20.9	101.1 ± 23.3*∗∗*	108.4 ± 15.5*∗∗*	116.2 ± 9.8*∗∗*
No. of nuclei	191.6 ± 49.2	219.2 ± 55.5*∗*	223.4 ± 49.8*∗∗*	209.7 ± 54.7*∗∗*
GH	0.020 ± 0.010	0.030 ± 0.013*∗∗*	0.030 ± 0.011*∗∗*	0.031 ± 0.019*∗∗*
IGF-I	0.036 ± 0.025	0.045 ± 0.027	0.054 ± 0.035*∗∗*	0.045 ± 0.023
Akt	0.167 ± 0.008	0.022 ± 0.015*∗*	0.024 ± 0.011*∗*	0.027 ± 0.012*∗∗*
AT-1	0.021 ± 0.011	0.021 ± 0.012	0.030 ± 0.020*∗∗*	0.026 ± 0.010
ET-1	0.019 ± 0.011	0.020 ± 0.012	0.026 ± 0.016*∗*	0.016 ± 0.010
ErK1/2	0.039 ± 0.20	0.021 ± 0.012*∗∗*	0.024 ± 0.011*∗∗*	0.017 ± 0.010*∗∗*^a^

*∗p* < 0.05. *∗∗p* < 0.01. *ap* < 0.05 compared to thes exercise group. GH: growth hormone; IGF-I: insulin-like growth factor-I; Akt: also known as protein kinase B (PKB); AT-1: angiotensin-II receptor type 1; ET-1: endothelin-1; Erk1/2: extracellular signal-regulated kinases 1/2.

## Data Availability

The datasets used and analyzed during the current study are available from the corresponding author on reasonable request.

## Abbreviations

Ns: *Nigella sativa*
N.s.: *Nigella sativa*-fed group
Ex.: Exercise-trained group
N.s.Ex.: *Nigella sativa*-fed exercise-trained group
GH: Growth hormone
IGF-1: Insulin-like growth factor-I
PI3K: Phosphoinositide 3-kinase
Akt: Also known as protein kinase B (PkB)
GPCR: G-protein-coupled receptors
AT-1: Angiotensin-II receptor type 1
ET-1: Endothelin-1
MAPK: Mitogen-activated protein kinase
Erk1/2: Extracellular signal-regulated kinases 1/2
ANOVA: Analysis of variance
LSD: Least significant difference
SD: Standard deviation.
